# Inflammatory cell-mediated tumour progression and minisatellite mutation correlate with the decrease of antioxidative enzymes in murine fibrosarcoma cells

**DOI:** 10.1038/sj.bjc.6690060

**Published:** 1999-02

**Authors:** F Okada, K Nakai, T Kobayashi, T Shibata, S Tagami, Y Kawakami, T Kitazawa, R Kominami, S Yoshimura, K Suzuki, N Taniguchi, O Inanami, M Kuwabara, H Kishida, D Nakae, Y Konishi, T Moriuchi, M Hosokawa

**Affiliations:** Laboratory of Pathology, Cancer Institute, Sapporo, 060-8638, Japan; Department of Oral Surgery, Health Science, University of Hokkaido, Ishikari, 061-0293, Japan; First Department of Medicine, Hokkaido University School of Medicine, Sapporo, 060-8638, Japan; First Department of Biochemistry, Niigata University School of Medicine, Niigata, 951-8510, Japan; Department of Molecular Life Science (Cell Biology), Tokai University School of Medicine, Isehara, 259-1193, Japan; Laboratory of Cell Biology, Hokkaido University School of Medicine, Sapporo, 060-8638, Japan; Department of Biochemistry, Osaka University Medical School, Osaka, 565-0871, Japan; Department of Environmental Veterinary Sciences, Graduate School of Veterinary Medicine, Sapporo, 060-0818, Japan; Department of Oncological Pathology, Cancer Center, Kashihara, 634-8521, Japan

**Keywords:** tumour progression, inflammation, antioxidative enzymes, active oxygen species, minisatellite instability

## Abstract

We isolated six clones of weakly tumorigenic fibrosarcoma (QR) from the tumorigenic clone BMT-11 cl-9. The QR clones were unable to grow in normal C57BL/6 mice when injected s.c. (1 × 10^5^ cells). However, they formed aggressive tumours upon co-implantation with a ‘foreign body’, i.e. a gelatin sponge, and the rate of tumour take ranged from 8% to 58% among QR clones. The enhanced tumorigenicity was due to host cell-mediated reaction to the gelatin sponge (inflammation). Immunoblot analysis and enzyme activity assay revealed a significant inverse correlation between the frequencies of tumour formation by QR clones and the levels of manganese superoxide dismutase (Mn-SOD, *P*<0.005) and glutathione peroxidase (GPχ, *P*<0.01) in the respective tumour clones. Electron spin resonance (ESR) revealed that superoxide-scavenging ability of cell lysates of the QR clone with high level of Mn-SOD was significantly higher than that with low level of the antioxidative enzyme in the presence of potassium cyanide, an inhibitor for copper–zinc superoxide dismutase (CuZn-SOD) (*P*<0.001). Minisatellite mutation (MSM) induced by the inflammatory cells in tumour cells were investigated by DNA fingerprint analysis after QR clones had been co-cultured with gelatin-sponge-reactive cells. The MSM rate was significantly higher in the subclones with low levels of Mn-SOD and GPχ (*P*<0.05) than in the subclones with high levels of both enzymes. The MSM of the subclones with low levels of both enzymes was inhibited in the presence of mannitol, a hydroxyl radical scavenger. The content of 8-hydroxydeoxyguanosine (8-OHdG) by which the cellular DNA damage caused by active oxygen species can be assessed was significantly low in the tumours arising from the QR clone with high levels of Mn-SOD and GPχ even if the clone had been co-implanted with gelatin sponge, compared with the arising tumour from the QR clone with low levels of those antioxidative enzymes (*P*<0.001). In contrast, CuZn-SOD and catalase levels in the six QR clones did not have any correlation with tumour progression parameters. These results suggest that tumour progression is accelerated by inflammation-induced active oxygen species particularly accompanied with declined levels of intracellular antioxidative enzymes in tumour cells. © 1999 Cancer Research Campaign


*British Journal of Cancer* (1999) **79**(3/4), 377–385
© 1999 Cancer Research Campaign
Article no. bjoc.1998.0060

### Inflammatory cell-mediated tumour progression and minisatellite mutation correlate with the decrease of antioxidative enzymes in murine fibrosarcoma cells

**F Okada[1], K Nakai[1], T Kobayashi[1], T Shibata[2], S Tagami[3], Y Kawakami[3], T Kitazawa[4], R Kominami[4], S Yoshimura[5], K Suzuki[7], N Taniguchi[7], O Inanami[8], M Kuwabara[8], H Kishida[9], D Nakae[9], Y Konishi[9], T Moriuchi[6] and M Hosokawa[1]**

[1]Laboratory of Pathology, Cancer Institute, Hokkaido University School of Medicine, Sapporo, 060-8638, Japan; [2]Department of Oral Surgery, Health Science, University of Hokkaido, Tobetsu, Ishikari, 061-0293, Japan; [3]First Department of Medicine, Hokkaido University School of Medicine, Sapporo, 060-8638, Japan; [4]First Department of Biochemistry, Niigata University School of Medicine, Niigata, 951-8510, Japan; [5]Department of Molecular Life Science (Cell Biology), Tokai University School of Medicine, Isehara, 259-1193, Japan; [6]Laboratory of Cell Biology, Hokkaido University School of Medicine, Sapporo, 060-8638, Japan; [7]Department of Biochemistry, Osaka University Medical School, Osaka, 565-0871, Japan; [8]Department of Environmental Veterinary Sciences, Graduate School of Veterinary Medicine, Hokkaido University, Sapporo, 060-0818, Japan; [9]Department of Oncological Pathology, Cancer Center, Nara Medical University, Kashihara, 634-8521, Japan

**Summary** We isolated six clones of weakly tumorigenic fibrosarcoma (QR) from the tumorigenic clone BMT-11 cl-9. The QR clones were unable to grow in normal C57BL/6 mice when injected s.c. ($1 \times 10^5$ cells). However, they formed aggressive tumours upon co-implantation with a 'foreign body', i.e. a gelatin sponge, and the rate of tumour take ranged from 8% to 58% among QR clones. The enhanced tumorigenicity was due to host cell-mediated reaction to the gelatin sponge (inflammation). Immunoblot analysis and enzyme activity assay revealed a significant inverse correlation between the frequencies of tumour formation by QR clones and the levels of manganese superoxide dismutase (Mn-SOD, $P<0.005$) and glutathione peroxidase (GPχ, $P<0.01$) in the respective tumour clones. Electron spin resonance (ESR) revealed that superoxide-scavenging ability of cell lysates of the QR clone with high level of Mn-SOD was significantly higher than that with low level of the antioxidative enzyme in the presence of potassium cyanide, an inhibitor for copper–zinc superoxide dismutase (CuZn-SOD) ($P<0.001$). Minisatellite mutation (MSM) induced by the inflammatory cells in tumour cells were investigated by DNA fingerprint analysis after QR clones had been co-cultured with gelatin-sponge-reactive cells. The MSM rate was significantly higher in the subclones with low levels of Mn-SOD and GPχ ($P<0.05$) than in the subclones with high levels of both enzymes. The MSM of the subclones with low levels of both enzymes was inhibited in the presence of mannitol, a hydroxyl radical scavenger. The content of 8-hydroxydeoxyguanosine (8-OHdG) by which the cellular DNA damage caused by active oxygen species can be assessed was significantly low in the tumours arising from the QR clone with high levels of Mn-SOD and GPχ even if the clone had been co-implanted with gelatin sponge, compared with the arising tumour from the QR clone with low levels of those antioxidative enzymes ($P<0.001$). In contrast, CuZn-SOD and catalase levels in the six QR clones did not have any correlation with tumour progression parameters. These results suggest that tumour progression is accelerated by inflammation-induced active oxygen species particularly accompanied with declined levels of intracellular antioxidative enzymes in tumour cells.

**Keywords:** tumour progression; inflammation; antioxidative enzymes; active oxygen species; minisatellite instability

Cancer development and its progression are considered to be the results of a series of distinct steps (Foulds, 1965; Pitot, 1986, 1989; Heppner and Dorcey, 1988). The particular term 'tumour progression' refers to the conversion of a 'benign' tumour into a 'malignant' one, the latter of which has acquired more aggressive properties manifested by rapid growth, invasiveness and metastatic ability as well as increased genetic instability leading to multiple genetic alterations (Foulds, 1965; Pitot, 1989). Because the last is thought to have the driving force of the process, it is crucial to identify the possible intracellular and extracellular mechanisms that accelerate tumour progression.

*Received 9 January 1998*
*Revised 7 July 1998*
*Accepted 13 July 1998*

*Correspondence to:* F Okada, Laboratory of Pathology, Cancer Institute, Hokkaido University School of Medicine, Kita-15, Nishi-7, Kita-ku, Sapporo, 060-8638, Japan

Leucocytes and other phagocytic cells exert their cytotoxic effect on bacteria, parasites and tumour cells by producing a variety of active oxygen species such as superoxide anion ($O_2^-$), hydrogen peroxide, nitric oxide and $OCl^-$ (Weitzman and Gordon, 1990; Ischiropoulos et al, 1992). These active oxygen species not only kill target cells but also lead to damage important biomolecules including DNA of the cell (Yamashina et al, 1986; Shacter et al, 1988). Thereby, active oxygen species are considered as one of the major contributors to carcinogenesis (Lewis and Adams, 1987; Ames et al, 1993). A recent report suggested that active oxygen species derived from chronic inflammatory cells may be a primary factor in the development of up to one-third of all cancers (Ames et al, 1993). Despite the apparent epidemiological association of inflammation with cancer, the relationship between inflammation and tumour progression is not fully understood because of a lack of a suitable animal model.

A clone (QR-32) derived from murine fibrosarcoma is poorly tumorigenic when injected s.c. into syngeneic C57BL/6 mice.

However, this cell line manifests highly malignant phenotypes once it has grown in vivo after being co-implanted with a 'foreign body' such as gelatin sponge (Okada et al, 1992). Our previous studies revealed that the co-implantation of the gelatin sponge with tumour cells induced inflammation at the site of tumour growth, and that the reactive inflammatory cells participated in the progression of QR-32 cells (Okada et al, 1992). We speculated that various active oxygen species produced by such inflammatory cells contributed to the tumour-promoting process with their mutagenic effects on tumour cells (Okada et al, 1992, 1994). In fact, tumour cell lines established from the tumours arising in mice with QR cells co-implanted with foreign bodies such as gelatin sponge or plastic plate acquired stable malignant phenotypes (Young et al, 1991; Okada et al, 1992, 1993).

Because the rates of tumour take after co-implantation with gelatin sponge varied among QR clones, we suspected there may be differences in susceptibility to endogenous progression-accelerating effects of active oxygen species. This study was designed to prove the hypothesis. We compared the expression and the activity levels of several antioxidative enzymes in a series of QR clones and confirmed the difference in superoxide-scavenging ability between the two QR clones, which were sensitive and resistant to inflammatory cell-induced progression, by electron spin resonance (ESR) techniques. We also examined minisatellite mutation (MSM) frequencies in the two QR clones by DNA fingerprint analysis using the Pc-1 minisatellite (MS) probe. We further examined 8-hydroxydeoxyguanosine (8-OHdG) formation between the two QR tumour tissues after co-implantation of gelatin sponge. Our results suggest that the decrease in the antioxidative capacity and the increase in the sensitivity to MSM induced by inflammatory cells both correlate with tumour progression.

### MATERIALS AND METHODS

### Animals

Female C57BL/6 mice between 2 and 4 months of age were obtained from Japan SLC.

### Tumour cells and culture conditions

The origin and characteristics of the tumour cells used have been described previously (Ishikawa et al, 1987*a*, 1987*b*; Okada et al, 1990). Briefly, BMT-11, a transplantable fibrosarcoma, was induced in a C57BL/6 mouse by 3-methyl-cholanthrene, and the tumorigenic clone BMT-11 cl-9 was subsequently isolated by limiting dilution (Ishikawa et al, 1987*a*). BMT-11 cl-9 cells exposed in vitro to quercetin gave rise to a number of random subclones. They spontaneously regress when injected into syngeneic mice. These variants were named 'QR clones' representing 'quercetin-induced regressor tumour' (Ishikawa et al, 1987*b*). Experiments on the tumour cell lines and the co-culture were carried out in monolayer cultures in Eagle's minimum essential medium (MEM) that contained 8% fetal bovine serum (FBS), sodium pyruvate, non-essential amino acids and L-glutamine, at 37°C, in a humidified 5% carbon dioxide/95% air mixture (Okada et al, 1992).

### Procedures for co-implantation of QR clones with gelatin sponge and preparation of gelatin-sponge-reactive cells

A piece of gelatin sponge (10 × 5 × 3 mm) was inserted into a surgically created subcutaneous pocket in recipient mice. Various 'QR clone' cells ($1 \times 10^5$ 0.1 $ml^{-1}$) were individually injected into the site of the preinserted gelatin sponge (Okada et al, 1992). The culture cell lines established from tumours that arose after co-implantation of QR-32 cells with gelatin sponge were designated QR32sP representing 'progressor tumour cells after co-implantation with sponge'. Because these QRsP tumour lines were cultured for at least 2 weeks, the QRsP tumour lines were not contaminated with any (or even negligible) inflammatory cells. As we did not observe any tumour in the mice which received gelatin sponge alone up to 12 months after implantation, we considered that the arising tumours were derived from the implanted QR clones and were not from transformation of host cells due to the gelatin sponge implantation. Average tumour diameters were measured twice weekly with vernier calipers. Peritoneal exudate formed in the mice which had been implanted with a gelatin sponge into the peritoneal cavity 5 days before it was collected, and cells isolated from the exudate were used as 'gelatin-sponge-reactive cells' (Okada et al, 1992). The average cellular composition of the gelatin-sponge-reactive cells was around 60% lymphocytes, 25% polymorphonuclear cells, 10% macrophages/monocytes and less than 2% of fibroblast-like cells as examined histologically.

### Procedures for co-culture of QR clones with gelatin-sponge-reactive cells

QR-32 cells or QR-29 cells ($1 \times 10^4$) were co-cultured with $1 \times 10^6$ gelatin-sponge-reactive cells in 24-well plastic plates (Falcon) in 2 ml of the medium for 48 h with or without the hydroxyl radical scavenger mannitol ($5 \times 10^{-2}$ M, Sigma). The medium was changed every day and the tumour cells grew to a subconfluent state 5–6 days after plating. They were detached and expanded to be used for single cell cloning.

### Cloning of tumour cells

QR-32 and QR-29 cells after co-culture with or without gelatin-sponge-reactive cells were seeded into 96-well flat-bottom microplates (Iwaki, Japan) at the density of 0.2 cells per well. All of the resultant cell clones were considered to have tumour origins, as determined by morphological features. The individual tumour clones were expanded and subjected to DNA extraction procedures for DNA fingerprinting.

### Minisatellite (MS) DNA probe

The pPc-1 plasmid, which contains tandem repeats of the 5′-GGGCA-3′ sequence flanked with locus-specific polymorphic sequences, was digested with *Pst*I and *Eco*RI, and the fragment which was 1.0 kb in length was purified. The Pc-1 MS fragment was labelled with [$\alpha$-$^{32}$P]dCTP by the use of Random Primer DNA Labelling Kit version 2.0 (Takara, Japan).

### DNA extraction and DNA fingerprint analysis of tumour cells

High molecular weight cellular DNA was extracted from the cloned tumour cells as previously reported (Takada et al, 1992). A 5-μg sample of each *Hae* III-digested DNA was electrophoresed through 1.25% agarose gel in 1× TAE buffer (40 mM tris-HCl, 40 mM acetic acid, 1 mM EDTA, pH 8.0) at 2 V $cm^{-1}$ for 20–24 h, and then the DNA was transferred to a nylon filter membrane

 © Cancer Research Campaign 1999

(Amersham) according to the method of Southern (1975). Prehybridization was carried out in 4 × SSC, 1% SDS, 10 mM tris-HCl, pH 7.5, and 10 μg ml$^{-1}$ yeast RNA at 60°C for 2 h. The membranes were then hybridized in the same prehybridization buffer containing $^{32}$P-labelled Pc-1 probe for 16–24 h at 65°C. The membranes were washed in 1 × SSC with 1% SDS at 65°C for 30 min and in 0.2 × SSC with 1% SDS at 65°C for 30 min, and exposed to radiographic film (X-Omat, Kodak) at –80°C for 1–7 days (Takada et al, 1992; Kitazawa et al, 1994; Nakagawa et al, 1997).

### DNA isolation and determination of 8-hydroxy-deoxyguanosine (8-OHdG) in tumour tissue DNA

DNA from the tumour tissue was isolated with a Sepagene kit (Sanko Junyaku, Japan) as described previously (Nakae et al, 1995). The levels of 8-OHdG were quantitated by the HPLC/ECD method of Kasai et al (Kasai et al, 1987). The 8-OHdG levels were calculated by calibration against curves from high-performance liquid chromatography (HPLC) and electro chemical detector (ECD) runs of standard authentic 8-OHdG (Yamasa Shoyu, Japan) and dG (2′-deoxyguanosine, Sigma) and were expressed as the numbers of 8-OHdG formed per $10^5$ total dG nucleosides.

### Immunoblotting for antioxidative enzymes

Lysates of the cultured QR clones were prepared in Laemmli's buffer (Laemmli, 1970) after ultrasound sonication and centrifugation at 15 000 r.p.m. for 15 min. Ten micrograms of protein from the cell extracts was transferred to a nitrocellulose membrane filter (Bio-Dot Blotting Media) with the use of slot blotting apparatus (Bio-Dot SF). The filters were preincubated for 2 h with 3% bovine serum albumin (BSA) in phosphate-buffered saline (PBS) containing 0.1% Tween-20 (T-PBS). The filters were incubated with antibodies specific to manganese superoxide dismutase (Mn-SOD), copper–zinc superoxide dismutase (CuZn-SOD) (Suzuki et al, 1991*a*) and Seleno-GPx (Yoshimura et al, 1980), respectively, for 1 h at room temperature. After five washes with T-PBS, the filter was incubated with peroxidase-conjugated goat anti-rabbit IgG antiserum (Binding Site) for 1 h at room temperature. After five washes of the filter with T-PBS, the specific protein–antibody reaction was detected by the enhanced chemiluminescence (ECL) detection system (Amersham). The intensities of individual bands were semi-quantified by densitometer and indicated as integrated OD (Image Quant, Molecular Dynamics, Japan). Each membrane was stained with 0.1% Coomassie brilliant blue solution (Bio Rad, R-250) and equivalance of the loading protein was confirmed in each slot.

### Assay for enzymatic activity

The cultured tumour cells were washed three times with PBS, collected with a rubber policeman, treated by freezing and thawing in PBS and sonicated on ice four times, for 15 s each, by using a sonicator at the intensity of 4 (Tomy Seiko, Japan). Their homogenates were centrifuged at 3000 r.p.m. for 10 min and the resulting supernatant was used for enzymatic assay. Superoxide dismutase (SOD) activity was measured by the nitroblue tetrazolium (NBT) reduction method (Beauchamp and Fridovich, 1971; Li et al, 1995), with slight modifications. The Mn-SOD activity was examined at 30°C in 1 ml of 20 mM sodium carbonate buffer, pH 10.0, containing 0.1 mM EDTA, 0.2 mM xanthine, 12 μM NBT and 1.95 mU xanthine oxidase. Mn-SOD activity was determined as the remaining SOD activity after addition of 2 mM potassium cyanide. The reaction was measured by a spectrometer at 560 nm. The amount of enzyme reducing NBT by 50% was defined as one unit of SOD activity. Catalase activity was measured as described previously (Beers and Sizer, 1952; Cohen et al, 1970) with slight modifications. The decomposition of hydrogen peroxide was recorded at 230 nm on a chart recorder for 1 min at 37°C. GPx activity was determined according to the method of Beutler (1984) with a slight modification by using a β-buthyl hydroperoxide as substrate. One unit of enzyme activity was defined as 1 μmol NADPH oxidized min$^{-1}$ at 37°C. The enzyme activities of Mn-SOD, CuZn-SOD, catalase and GPx were expressed as units mg$^{-1}$ protein. Protein concentration was estimated by the method of Lowry et al (1951) using bovine serum albumin (BSA) as standard.

### Superoxide-scavenging activities of QR clones

The hypoxanthine-xanthin oxidase (HPX-XOD) system was used for generating superoxide. The spin-trapping agent used was 5,5-dimethyl-1-pyrroline-*N*-oxide (DMPO), which forms secondary radicals (spin adduct) with superoxide. The superoxide-scavenging ability of QR clone lysates was measured according to the previously described method (Kuwabara et al, 1996). A mixed solution of 80 μl of 0.1 M phosphate buffer (pH 7.4), 50 μl of 1 mM HPX (Sigma), 50 μl of 400 mM DMPO (Sigma) solution and 10 μl (10 μg protein) of the sample lysates [or 10 μl of 0.1 M phosphate buffer (pH 7.4) for control] was prepared. As soon as 10 μl of XOD (60 mU ml$^{-1}$, Sigma) was added, the solution was transferred to a quartz flat cell (Labotec) for ESR measurements. The ESR spectra were recorded on a JES-RE1X spectrometer equipped with an Esprit-425 computer system (Joel, Japan). Measurements were carried out at room temperature under the following conditions: magnetic field, 334.6 ± 10.0 mT; the field modulation, 100 kHz with an amplitude of 0.1 mT; indicated microwave power, 10.0 mW. The ESR signal intensity was estimated by measuring the peak-to-peak height at $M_1 = +1$. The maximum ESR signal intensities obtained by the addition of PBS with and without potassium cyanide were rated as 100% (control), respectively, and the ESR signal intensities obtained by the addition of cell lysates of QR clones were expressed as percentages of the control.

### Statistical analysis

The significance of the correlation in the susceptibility to tumour progression and the contents of antioxidative enzymes in QR clones were analysed and plotted with the use of Kaleidagraph 3.0 software (Synergy Software) and was evaluated by regression analysis. The differences in the subcutaneous tumour, lung metastasis incidences and the MSM frequencies were calculated by $\chi^2$ test. The differences in ESR signal, the differences in antioxidative enzyme activity and the differences in the 8-OHdG formation among QRsP tumours were calculated by Student's *t*-test.

### RESULTS

### Growth of QR regressor clones in syngeneic mice after co-implantation with gelatin sponge

None of six QR (i.e. quercetin-induced regressor tumour) clones was able to grow in normal syngeneic mice when 1 × $10^5$ cells

© Cancer Research Campaign 1999 

**Table 1** Growth of QR regressor clones in syngeneic C57BL/6 mice after co-implantation with gelatin sponge

| QR clones | No. of mice with tumours/ no. of mice injected (%) Gelatin sponge implantation[a] Yes | No | Average latency period (days) |
|---|---|---|---|
| QR-32 | 18/31[c] (58) | 0/13 (0) | 11.1±2.0 |
| QR-18 | 8/22[d] (36) | 0/10 (0) | 11.9±2.7 |
| QR-20 | 7/23[d] (30) | 0/11 (0) | 11.6±2.8 |
| QR-12 | 5/22[e] (23) | 0/8 (0) | 12.2±3.2 |
| QR-19 | 4/21[e] (19) | 0/10 (0) | 14.5±3.3 |
| QR-29 | 2/25[e] (8) | 0/13 (0) | 12.0±2.8 |
| None[b] | 0/20 (0) | – | – |

[a]Cells of QR clone ($1 \times 10^5$) were s.c. co-implanted with gelatin sponge; [b]Mice were inserted s.c. with gelatin sponge alone and examined for 12 months, but no tumours were observed. In contrast, all tumours arose within 20 days after QR clone s.c. co-implanted with gelatin sponge; [c]*P*<0.005; [d]*P*<0.05 vs animals injected with original QR clone cells alone; e, not significant. A summary of three separate experiments.

were injected s.c. as a single-cell suspension (Table 1). However, all of those clones formed lethal tumours after co-implantation with gelatin sponges. The respective rates of tumour take were 58% (for QR-32), 36% (QR-18), 30% (QR-20), 23% (QR-12), 19% (QR-19) and 8% (QR-29). We have previously observed that one of the clones (QR-32) was able to grow in mice after implantation of tumour cells in mixture with gelatin-sponge-induced inflammatory cells (Okada et al, 1992). Thus, inflammatory cells reacting to the gelatin sponge may directly promote tumorigenicity of QR clones. The cell lines established from tumours of individual QR clones were designated as QRsP (i.e. progressor tumour after co-implantation of QR cells with gelatin sponge).

### QRsP tumour cell lines' acquisition of malignant properties

Table 2 shows biological characteristics of established QRsP tumour cell lines with their original QR clones respectively. All QRsP tumour cell lines except QR29sP-2 grew in vivo without further co-implantation with gelatin sponge upon injection s.c. of $2 \times 10^5$ cells. The QRsP tumour lines metastasized to the lungs at high frequencies in contrast to their parental QR counterparts, in parallel with their greater tumorigenicity. We considered that the tumours arising at the co-implantation sites were found to be composed of more malignant cells than those of original inoculum because the established tumour lines from the arising tumours (QRsP) had acquired malignant phenotypes, i.e. enhanced subcutaneous tumorigenicity and lung metastatic ability compared with the original QR clones.

### Inverse correlation between the tendency to progress and the content of antioxidative enzymes in QR clones

We previously reported that inflammatory cells reactive to gelatin sponge are responsible for the progression of QR-32 cells, and proposed that active oxygen species produced by inflammatory cells may be involved in this process (Okada et al, 1992). Therefore, we examined the amount of antioxidative enzymes in QR clones. As shown in Figure 1, a significant inverse correlation is observed between the contents of immunoreactive proteins of

**Table 2** Acquisition of malignant properties of tumour lines (QRsP) derived from QR clones co-implanted with gelatin sponge

| Cells[a] | Subcutaneous injections: No. of mice with tumours/ no. of mice injected | Intravenous injections[e]: No. of mice with lung metastasis/no. of mice injected | No. of colonies per mouse lung[j] |
|---|---|---|---|
| QR-32 | 0/13 | 1/11 | 0,0,0,0,0,0,0,0,0,0,2 |
| QR32sP-1 | 2/5[b] | 8/8[f] | 20,32,34,77,85,88,115,164 |
| QR32sP-2 | 5/5[c] | 8/8[f] | >150,>150,>150,>150,>150,>150,>150,>150 |
| QR32sP-3 | 3/5[d] | 8/8[f] | >150,>150,>150,>150,>150,>150,>150,>150 |
| QR32sP-4 | 5/5[c] | 6/7[g] | 0,10,27,29,62,69,77 |
| QR-18 | 0/10 | 0/6 | 0,0,0,0,0,0 |
| QR18sP-1 | 5/5[c] | 5/5[f] | 10,10,13,26,26 |
| QR18sP-2 | 4/5[c] | 3/5[h] | 0,0,3,6,19 |
| QR18sP-3 | 2/5[b] | 5/5[f] | 7,9,22,25,30 |
| QR-20 | 0/11 | 0/6 | 0,0,0,0,0,0 |
| QR20sP-1 | 1/5[b] | 4/5[g] | 0,10,13,14,49 |
| QR20sP-2 | 5/5[c] | 4/5[g] | 0,1,1,15,18 |
| QR20sP-3 | 5/5[c] | 4/4[g] | 5,8,23,40 |
| QR-12 | 0/8 | 0/5 | 0,0,0,0,0 |
| QR12sP-1 | 4/5[d] | 4/5[g] | 0,3,4,16,48 |
| QR12sP-2 | 4/5[d] | 4/4[g] | 3,5,40,45 |
| QR-29 | 0/13 | 0/7 | 0,0,0,0,0,0,0 |
| QR29sP-1 | 3/5[d] | 4/5[g] | 0,3,13,14,23 |
| QR29sP-2 | 0/5[b] | 2/5[i] | 0,0,0,2,2 |

[a]Each regressor clone cell was co-implanted s.c. with gelatin sponges ($1 \times 10^5$ cells per sponge); culture cell lines (QRsP) were separately established from tumours which had arisen in each mouse; normal mice were injected s.c. with $2 \times 10^5$ cells from either progressor tumour cells or the original QR clone cells; [b]not significant; [c]*P*<0.01; [d]*P*<0.05 vs original QR clone cells. In a separate experiment, mice were i.v. injected with $1 \times 10^6$ of each type of the cells. [e]Nineteen days later, the mice were sacrificed and the metastatic nodules on the lung surface were counted macroscopically. The incidences of lung metastasis: [f]*P*<0.001; [g]*P*<0.01; [h]*P*<0.05; [i]not significant vs original QR clone cells; [j]each value represents the number of colonies per mouse lung. A summary of two to three separate experiments.

 *© Cancer Research Campaign 1999*

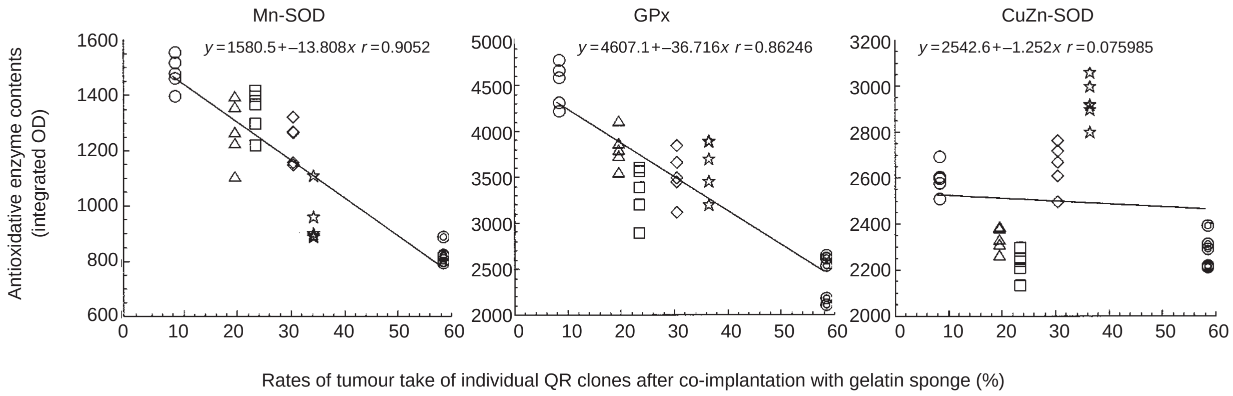

**Figure 1** Inverse correlation between the contents of manganese superoxide dismutase or glutathione peroxidase and the progression frequency of QR clones. Cell protein/slot (10 µg) was analysed with the use of the antibodies specific to Mn-SOD, CuZn-SOD and GPx respectively. Antibody-specific proteins were visualized by the enhanced chemiluminescence detection (ECL) system and the individual bands were semiquantified by densitometer. Equivalance of the loading protein was confirmed by staining the membrane with 0.1% Coomassie brilliant blue solution. Data indicates representative results of at least three experiments with similar results. The frequencies of progression (%) of QR clones represented by abscissa corresponding to the percentage of the tumorigenicity of each QR clone after co-implantation with gelatin sponge in Table 1. ◎, QR-32; ☆, QR-18; ◇, QR-20; □, QR-12; △, QR-19 and ○, QR-29

**Table 3** Activities of manganese, copper and zinc superoxide dismutase, catalase and glutathione peroxidase in QR clones

| QR clones | Antioxidative enzyme activities $mg^{-1}$ protein | | | | Tumorigenicity[a] (rate of tumour take, %) |
|---|---|---|---|---|---|
| | Mn-SOD (U) | CuZn-SOD (U) | GPx (mU) | Catalase (U) | |
| QR-32 | 6.1±0.7* | 33.7±1.0* | 119.2±1.2* | 22.3±0.6** | 58 |
| QR-29 | 12.6±0.2* | 52.2±1.7* | 191.1±3.7* | 23.1±1.1** | 8 |

[a]$P<0.001$; [b]not significant. Statistical difference of each antioxidative enzyme activity was calculated between QR-32 and QR-29 cells. The tumorigenicity of the QR clones promoted by the co-implantation with gelatin sponge was indicated. See Table 1.

manganese superoxide dismutase (Mn-SOD) or glutathione peroxidase (GPx) and the rates of tumour take, i.e. frequencies of progression of QR clones (Mn-SOD: r=–0.905, $P<0.005$; GPx: r=–0.862, $P<0.01$). In contrast, no such inverse correlation was found in the content of CuZn-SOD. We confirmed the equivalence of the loaded protein in each slot by staining the membrane with 0.1% Coomassie brilliant blue solution after observing antioxidative enzyme detections (data not shown). We also measured the activities of Mn-SOD, CuZn-SOD, GPx and catalase in QR-32 and QR-29 cells, and found that the decreased activity of Mn-SOD and GPx correlated with the higher tumour take rates when QR clones were co-implanted with gelatin sponge (Table 3). Figure 2 shows that the electron spin resonance (ESR) techniques with 5,5-dimethyl-1-pyrroline-*N*-oxide (DMPO) revealed the superoxide-scavenging ability of the QR clones. The ESR signal of DMPO-OOH (DMPO spin adduct of superoxide anion; $a_N$=1.40 mT, $a_H^{\beta}$=1.13 mT and $a_H^{\gamma}$=0.14 mT) was observed after the addition of xanthine oxidase to the reaction mixture containing hypoxanthine and DMPO in the potassium phosphate buffer [Figure 2A(a)]. SOD (40 U $ml^{-1}$) completely inhibited the DMPO-OOH formation [Figure 2A(c)]. An addition of the cell lysates of QR-29 cells, in contrast to that of QR-32 cells, decreased the signal intensity of DMPO-OOH [Figure 2A (d and f)]. The difference in the signal intensity of DMPO-OOH was evident in the presence of potassium cyanide (1 mM) [Figure 2A (e and g)]. Proteins or enzymes with superoxide-scavenging ability in mammalian cells are known to be inhibited by cyanide ($CN^-$). CuZn-SOD, extracellular SOD and ceruloplasmin are inhibited by potassium cyanide, whereas

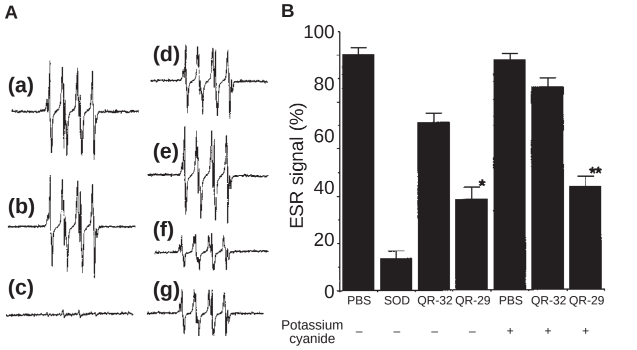

**Figure 2** ESR spectra of DMPO-OOH spin adducts formed in both HPX-XOD system plus DMPO and the cell lysate from either QR-32 or QR-29 cells. Typical ESR spectra were recorded with the spin trap DMPO on 10 µg of the protein obtained from QR-32 or QR-29 cells with or without adding potassium cyanide (1 mM). **A**: (**a**) PBS control; (**b**) PBS plus potassium cyanide; (**c**) PBS plus SOD (40 U $ml^{-1}$); (**d**) QR-32; (**e**) QR-32 plus potassium cyanide; (**f**) QR-29; (**g**) QR-29 plus potassium cyanide. **B**: Reduction in superoxide ($O_2^-$) formation after addition of QR-29 cell lysates. Values were expressed as percentage of ESR signals of PBS control with or without potassium cyanide and represent the means ± s.d. All measurements were in triplicate and were reproduced at least three times. *$P<0.05$; **$P<0.001$ compared with respective QR-32 cells

Mn-SOD is not. A summary of three independent experiments with similar results are shown in Figure 2B, confirming that QR-29 cells possessed significant highly superoxide-scavenging ability compared with QR-32 cells.

© Cancer Research Campaign 1999 

**Table 4** Minisatellite mutations (MSMs) induced in QR clones after co-culture with gelatin-sponge-reactive cells

| QR clones | Sponge-reactive cells | In the presence of mannitol | No. of subclones with MSMs/subclones examined (%) |
|---|---|---|---|
| QR-32 | No | No | 0/22 (0) |
| QR-32 | Yes | No | 5/23 (22)[a] |
| QR-32 | Yes | Yes | 1/22 (7)[b] |
| QR-29 | No | No | 0/20 (0) |
| QR-29 | Yes | No | 1/14 (7)[b] |
| QR-29 | Yes | Yes | 2/21 (12)[b] |

Each type of cell ($1 \times 10^4$ QR-32 or QR-29 cells, and $1 \times 10^6$ gelatin-sponge-reactive cells) were co-cultured in 24-well plastic plates in 2 ml of MEM medium with or without mannitol ($5 \times 10^{-2}$ M) for 48 h. [a]*P*<0.05; [b]not significant vs MSM observed in original QR clone without adding sponge-reactive cells and mannitol.

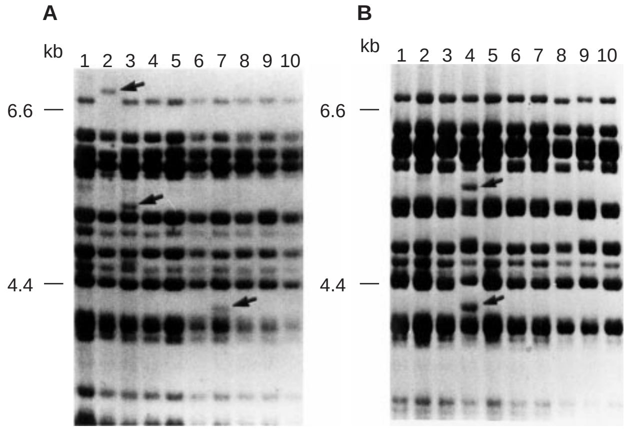

**Figure 3** MSMs induced in QR-32 and QR-29 cells after co-culture with gelatin-sponge-reactive cells as detected by DNA fingerprinting. QR cells ($1 \times 10^4$) were co-cultured with $1 \times 10^6$ gelatin-sponge-reactive cells for 48 h. The tumour cells were recloned and the individual subclones were examined for DNA fingerprinting. (**A**) Subclones obtained from QR-32 cells after co-culture with gelatin-sponge-reactive cells. (**B**) Subclones obtained from QR-29 cells after co-culture with gelatin-sponge-reactive cells. Lanes 1–10 indicate DNA fingerprints of individual subclones derived from QR-32 (**A**) or QR-29 (**B**) after co-culture with gelatin-sponge-reactive cells

### Minisatellite mutation (MSM) among QR clones after co-culture with gelatin-sponge-reactive cells

Frequencies of minisatellite mutation (MSM) induced in the QR-32 and QR-29 cells by gelatin-sponge-reactive cells are summarized in Table 4. In a separate experiment, flow cytometric analysis using an oxidation-sensitive fluorescent compound detected that production of intracellular peroxides by QR clones was increased after co-culture with gelatin-sponge-reactive cells (data not shown). No such induced MSMs were detected in 22 and 20 subclones of untreated QR-32 and QR-29 cells respectively. However, when the cells were co-cultured with gelatin-sponge-reactive cells, a significant increase in frequency of MSM was observed in 5 out of 23 (22%) QR-32 subclones (*P*<0.05) and 1 out of 14 (7%) QR-29 subclones (the latter not significant). Typical MSMs are shown by arrow heads in Figure 3. MSM rate, induced in QR-32 cells in the presence of gelatin-sponge-reactive cells, declined to 1 in 22 subclones (7%) when a hydroxyl radical scavenger, mannitol ($5\times10^{-2}$ M), was added.

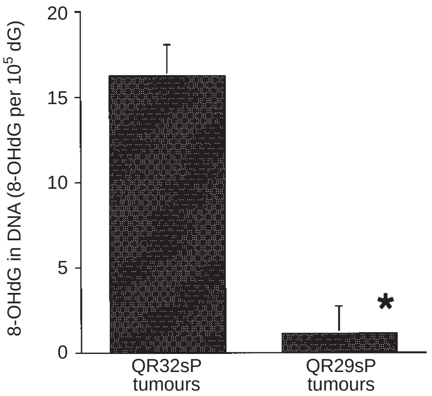

**Figure 4** 8-OHdG levels in tumour tissues obtained from QR32sP or QR29sP tumours. One $\times 10^5$ or QR-32 or QR-29 cells, respectively, were co-implanted with a gelatin sponge in C57BL/6 mice. Twenty-one days later, the arising tumours were removed and the DNA was isolated from individual tumour tissues and the 8-OHdG levels were measured. Each value represents the average from four QR32sP tumours or three QR29sP tumours ± s.d. **P*<0.001 compared with QR32sP tumours

### Inhibition of 8-hydroxyguanosine formation in the arising tumour tissue DNA obtained from QR-29 cells co-implanted with gelatin sponge

8-Hydroxyguanosine (8-OHdG) is a promutagenic DNA lesion produced by active oxygen species (Kasai and Nishimura, 1984; Shibutani et al, 1991). We examined the level of 8-OHdG in tumour tissues after co-implantation of QR-32 and QR-29, respectively, with gelatin sponge (Figure 4). We measured 8-OHdG formation in four QR32sP tumours and three QR29sP tumours. Mean numbers of 8-OHdG formed per $10^5$ total deoxyguanosine (dG) nucleosides in the QR32sP and QR29sP tumour tissues were 16.3 ± 1.8 and 1.1 ± 1.6 respectively. A significant decrease in the 8-OHdG formation was observed in QR29sP tumour tissues compared with QR32sP tumour tissues (*P*<0.001).

### DISCUSSION

In this study, we revealed that the susceptibility to undergo tumour progression induced by inflammatory cells and MSMs may depend on antioxidative enzyme levels in the tumour cells; i.e. the six mouse QR regressor clones exhibited different levels of tendency to progress after co-implantation with gelatin sponge that corresponded to the Mn-SOD and GPx contents. Because the QRsP tumour lines had stable malignant phenotypes in vivo (Table 2), the progression of QR clones was considered to be due to genetic alterations induced by gelatin-sponge-reactive cells.

Gelatin sponge is known to induce a classic inflammatory response; initially, polymorphonuclear (PMN) leucocytes infiltrate the sponges, which is followed by accumulation of macrophages/monocytes and other inflammatory cells (Ascher et al, 1979; Akporiaye and Kudalore, 1989; Middleton and Campbell, 1989). Stimulated phagocytic cells undergo a respiratory burst, and thus a variety of active oxygen species are released into the extracellular environment (Badway and Karnovsky, 1980). Of inflammatory oxidants generated by leucocytes, hydrogen peroxide is the species that diffuses freely into neighbouring cells (Chance et al, 1979) and reaches their nuclei unless they are enzymatically

 © Cancer Research Campaign 1999

degraded, or reacts with another cellular target. In mammalian cells, superoxide anion ($O_2^-$) is intracellularly produced in mitochondria whereas hydrogen peroxide is produced in mitochondria and peroxisomes during normal aerobic metabolism. As a defence system, cells must possess specific enzymes that eliminate the active oxygen species. Mitochondria contain their own Mn-SOD which catalyses dismutation of $O_2^-$ to molecular oxygen and hydrogen peroxide (Kawaguchi et al, 1989). Hydrogen peroxide produced by mitochondria and peroxisomes diffuses into the cytosol (Schreck and Baeuerle, 1991) where hydrogen peroxide is reduced by GPx to water. The classical intracellular GPx is mainly a cytosolic enzyme, which is also found in the mitochondrial matrix of mammalian cells (Flóhe, 1982).

We found a highly significant inverse correlation between intracellular contents of Mn-SOD or GPx and the rate of tumour take, i.e. frequencies of progression in a series of ‘regressor’ type QR clones. In contrast, contents and activities of CuZn-SOD and activity of catalase in the cells did not have any correlations with the frequencies of progression of QR clones. We further confirmed that superoxide-scavenging ability of the cell lysate from the QR clone with a high level of Mn-SOD was significantly higher than that with a low level of the antioxidative enzyme, by using ESR. Low activity of Mn-SOD may facilitate the generation of $O_2^-$ in mitochondria, and overproduced $O_2^-$ may be released into the cytosol where cytosolic CuZn-SOD dismutates $O_2^-$ into hydrogen peroxide. Because the $K_m$ of GPx is lower than that of catalase for hydrogen peroxide and catalase is localized mainly in peroxisomes, elevated levels of hydrogen peroxide in the cytosol are probably metabolized preferentially by GPx in vivo (Cohen and Hochstein, 1963). Therefore, the decreased activity of GPx may result in an accumulation of hydrogen peroxide in the cytosol. The toxicity of hydrogen peroxide in oxidative stress comes from the formation of a highly active oxygen species in the presence of suitable transitional metal catalysts by way of a Fenton-type reaction (Halliwell, 1987). This active oxygen species is most likely the hydroxyl radical ($^\bullet$OH), which can cause strand breakage or chemical modification of the bases in DNA (Schraufstatter et al, 1988; Aruoma et al, 1991). We showed evidence for such a scenario by measuring 8-OHdG formation, which is an adduct that results from the damage to DNA caused by active oxygen species (Kasai and Nishimura, 1984; Shibutani et al, 1991). Significantly high 8-OHdG formation was observed in the tumour tissues derived from the QR-32 cells co-implanted with gelatin sponge, which originally possessed low levels of antioxidative enzymes (Figure 4).

DNA fingerprint detects mutations that result from homologous recombination, DNA slippage during replication and mutagen exposures or irradiation (Jeffreys et al, 1985; Suzuki et al, 1991*b*; Kitazawa et al, 1994). This analysis supports our hypothesis that phenotypic changes of QR clones in vivo can be ascribed to MSMs induced by a combined effect of active oxygen species released from inflammatory cells and decreased antioxidative enzymes in QR clones. MSM occurred at high frequency in QR-32 cells co-cultured with gelatin-sponge-reactive cells; and those QR-32 cells had the lowest activities of Mn-SOD and GPx among the six QR clones. MSM frequency of QR-32 cells decreased in the presence of mannitol, a hydroxyl radical scavenger (Table 4). In contrast, QR-29 cells, which have higher activities of Mn-SOD and GPx than QR-32 cells, had less MSM when co-cultured with gelatin-sponge-reactive cells. These observations strongly suggest that MSM was induced by active oxygen radicals mediated by inflammatory cells. A report showed that alterations of MS occurred in somatic cells when they were exposed to various chemical carcinogens (Suzuki et al, 1991*b*; Kitazawa et al, 1994) or Okadaic acid, a tumour promoter (Nakagawa et al, 1997), with relatively high incidences of MSMs, which were detected with the same Pc-1 probe as we used in the present experiments. Recently, MSM has been detected in various human tumours (Thein et al, 1987; Matsumura and Tarin, 1992) as well as in experimental animal tumours (Ledwith et al, 1990, 1995); it is, thus, suspected that MS instability may be involved in oncogenesis.

Changes in DNA fingerprints could result from a number of processes, i.e. mutations of nucleotide sequence, mitotic recombination, loss of the chromosomal integrity and DNA slippage (Mitani et al, 1990; Hastie et al, 1990; Willard, 1990; Blackburn, 1991; Jeffreys et al, 1985). All these changes can be provoked by active oxygen species as a direct or indirect effect of the DNA damage. The Pc-1 MS sequence consists of tandem repeats of the GGGCA sequence and its homologous sequences are present throughout the mouse genome (Takada et al, 1992). Therefore, the changes in DNA fingerprints between the tandem repeats could provide markers for detecting somatic mutations. Although the MSM in the tandem repeat sequences may not directly account for tumour progression at present, the frequency of MSMs in this region is thought to reflect the genetic instability of other regions that regulate cell differentiation and growth, leading to tumour progression (Thein et al, 1987; Suzuki et al, 1991*b*; Takada et al, 1992).

It is well known that chronic inflammation is a major cause of carcinogenesis in the liver (Yu et al, 1991; Tabor and Kobayashi, 1992), colon (Choi and Zelig, 1994) and bladder (Rosin et al, 1994) and that the inflammatory processes are an important endogenous source of both active oxygen species and cytokines/growth factors. The endogenous source of cytokines/growth factors is known to be involved in the process of tumour progression (Films and Kerbel, 1993; Leek et al, 1994; Okada et al, 1994; Shimoda et al, 1994; Nagayasu et al, 1998). Moreover, recent data indicate that such cytokines/growth factors cause reduction of antioxidative enzyme expressions in normal cells, and set out a speculation that suppression of antioxidative enzymes may lead to greater susceptibility to the oxidative damage and to carcinogenesis (Kayanoki et al, 1994; Suemizu et al, 1994). We notice that some cancer tissues contain substantially high amounts of antioxidative enzymes compared with their normal counterparts (Taniguchi, 1992; Oberley and Oberley, 1997). These results indicate that the antioxidative enzyme imbalance leads to increase intracellular active oxygen species of tumour cells. Results obtained by using our experimental model indicate that tumour progression is accelerated by the active oxygen species produced by the inflammatory reaction at the tumour site and by the decrease in the primary antioxidative enzymes in tumour cells.

### ACKNOWLEDGEMENTS

This study was supported in part by a Grant-in-Aid for Cancer Research (06280119) from the Japanese Ministry of Health and Welfare. We thank Drs Janusz W Rak, Mark Micallef and Miss Masako Yanome for kindly revising the English descriptions.

### REFERENCES

Akporiaye ET and Kudalore MK (1989) Implantation of a gelatin sponge as a model for effector recruitment: tumor growth inhibition by T-lymphocytes recovered from a site of tumour rejection. *Cancer Immunol Immunother* **29**: 199–204

© Cancer Research Campaign 1999 

Ames BN, Shigenaga MK and Hagen TM (1993) Oxidants, antioxidants, and the degenerative diseases of aging. *Proc Natl Acad Sci USA* **90**: 7915–7922

Aruoma OI, Halliwell B, Gajewski E and Dizdaroglu M (1991) Copper-ion-dependent damage to the bases in DNA in the presence of hydrogen peroxide. *Biochem J* **273**: 601–604

Ascher NA, Eerguson RM, Hoffman R and Simmons RL (1979) Partial characterization of cytotoxic cells infiltrating spongematrix allografts. *Transplantation* **27**: 254–259

Badway JA and Karnovsky ML (1980) Active oxygen species and the functions of phagocytic leukocytes. *Annu Rev Biochem* **49**: 695–726

Beauchamp C and Fridovich I (1971) Superoxide dismutase: improved assay and an assay applicable to acrylamide gels. *Anal Biochem* **44**: 276–287

Beers Jr RF and Sizer IW (1952) A spectrophotometric method for measuring the breakdown of hydrogen peroxide by catalase. *J Biol Chem* **195**: 133–140

Beutler E (1984) A manual of biochemical methods. In *Red Cell Metabolism*, edn. 3. Beutler E (ed.) pp.72–136, Grune & Stratton: Philadelphia

Blackburn EH (1991) Structure and function of telomeres. *Nature* **350**: 569–573

Chance B, Sies H and Boveris A (1979) Hydroperoxide metabolism in mammalian organs. *Physiol Rev* **59**: 527–605

Choi PM and Zelig MP (1994) Similarity of colorectal cancer in Crohn's disease and ulcerative colitis: implications for carcinogenesis and prevention. *Gut* **35**: 950–954

Cohen G and Hochstein P (1963) Glutathione peroxidase: the primary agent for the elimination of $H_2O_2$ in erythrocytes. *Biochemistry* **2**: 1420–1428

Cohen G, Dembiec D and Marcus J (1970) Measurement of catalase activity in tissue extracts. *Anal Biochem* **34**: 30–38

Filmus J and Kerbel RS (1993) Development of resistance mechanisms to the growth-inhibitory effects of transforming growth factor-beta during tumor progression. *Curr Opin Oncol* **5**: 123–129

Flóhe L (1982) Glutathione peroxidase brought into focus. In *Free Radicals in Biology*. Pryor WA (ed.), pp. 223–254. Academic Press: New York

Foulds L (1965) Multiple etiologic factors in neoplastic development. *Cancer Res* **25**: 1339–1347

Halliwell B (1987) Oxidants and human disease: some new concepts. *FASEB J* **1**: 358–364

Hastie ND, Dempster M, Dunlop MG, Thompson AM, Green DK and Allshire RC (1990) Telomere reduction in human colorectal carcinoma and with aging. *Nature* **346**: 866–868

Heppner G and Dorcey L (1988) Macrophages and development of cancer. In *Macrophages and Cancer*. Heppner GH and Fulton AM (eds.), pp.197–208. CRC Press, Boca Raton, Florida

Ischiropoulos H, Zhu L and Beckman JS (1992) Peroxynitrite formation from macrophage-derived nitric oxide. *Arch Biochem Biophys* **298**: 446–451

Ishikawa M, Hosokawa M, Oh-hara N, Niho Y and Kobayashi H (1987*a*) Marked granulocytosis in C57Bl/6 mice bearing a transplanted BMT-11 fibrosarcoma. *J Natl Cancer Inst* **78**: 567–571

Ishikawa M, Okada F, Hamada J-I, Hosokawa M and Kobayashi H (1987*b*) Changes in the tumorigenic and metastatic properties of tumor cells treated with quercetin or 5-azacytidine. *Int J Cancer* **39**: 338–342

Jeffreys AJ, Wilson V and Thein SL (1985) Hypervariable 'minisatellite' regions in human DNA. *Nature* **314**: 67–73

Kasai H and Nishimura S (1984) Hydroxylation of deoxyguanosine at the C-8 position by ascorbic acid and other reducing agents. *Nucleic Acids Res* **12**: 2137–2145

Kasai H, Nishimura S, Kurokawa Y and Hayashi Y (1987) Oral administration of the renal carcinogen, potassium bromate, specifically produces 8-hydroxydeoxyguanosine in rat target organ DNA. *Carcinogenesis* **8**: 1959–1961

Kawaguchi T, Noji S, Uda T, Nakashima Y, Takeyasu A, Kawai Y, Takagi H, Tohyama M and Taniguchi N (1989) A monoclonal antibody against COOH$^-$ terminal peptide of human liver manganese superoxide dismutase. *J Biol Chem* **264**: 5762–5767

Kayanoki Y, Fujii J, Suzuki K, Kawata S, Matsuzawa Y and Taniguchi N (1994) Suppression of antioxidative enzyme expression by transforming growth factor-β1 in rat hepatocytes. *J Biol Chem* **269**: 15488–15492

Kitazawa T, Kominami R, Tanaka R, Wakabayashi K and Nagao M (1994) 2-Hydroxyamino-1-methyl-6-phenylimidazo[4,5-*b*]pyridine induction of recombinational mutations in mammalian cell lines as detected by DNA fingerprinting. *Mol Carcinog* **9**: 67–70

Kuwabara M, Inukai N, Inanami O, Miyake YI, Tsunoda N, Maki Y and Sato F (1996) Lipid peroxide levels and superoxide-scavenging abilities of sera obtained from hotbred (thoroughbred) horses. *J Vet Med Sci* **58**: 97–101

Laemmli UK (1970) Cleavage of structural proteins during the assembly of the head of bacteriophage T4. *Nature* **227**: 680–685

Ledwith BJ, Storer RD, Prahalada S, Manam S, Leander KR, van Zwieten MJ, Nichols WW and Bradley MO (1990) DNA fingerprinting of 7,12-dimethylbenz[a]anthracene-induced and spontaneous CD-1 mouse liver tumors. *Cancer Res* **50**: 5245–5249

Ledwith BJ, Joslyn DJ, Troilo P, Leander KR, Clair JH, Soper KA, Manam S, Prahalada S, van Zwieten MJ and Nichols WW (1995) Induction of minisatellite DNA rearrangements by genotoxic carcinogens in mouse liver tumors. *Carcinogenesis* **16**: 1167–1172

Leek RD, Harris AL and Lewis CE (1994) Cytokine networks in solid human tumors: regulation of angiogenesis. *J Leukocyte Biol* **56**: 423–435

Lewis JG and Adams DO (1987) Inflammation, oxidative DNA damage, and carcinogenesis. *Environ Health Perspect* **76**: 19–27

Li J-J, Oberley LW, St Clair DK, Ridnour LA and Oberley TD (1995) Phenotypic changes induced in human breast cancer cells by overexpression of manganese-containing superoxide dismutase. *Oncogene* **10**: 1989–2000

Lowry OH, Rosebrough NJ, Farr AL and Randall RJ (1951) Protein measurement with the folin phenol reagent. *J Biol Chem* **193**: 265–275

Matsumura Y and Tarin D (1992) DNA fingerprinting survey of various human tumors and their metastases. *Cancer Res* **52**: 2174–2179

Middleton MM and Campbell PA (1989) Functions of purified mouse neutrophils isolated from gelatin sponges. *J Leukocyte Biol* **46**: 461–466

Mitani K, Takahashi Y and Kominami R (1990) A GGCAGG motif in minisatellites affecting their germline instability. *J Biol Chem* **265**: 15203–15210

Nagayasu H, Hamada J-I, Nakata D, Shibata T, Kobayashi M, Hosokawa M and Takeichi N (1998) Reversible and irreversible tumor progression of a weakly malignant rat mammary carcinoma cell line by in vitro exposure to epidermal growth factor. *Int J Oncol* **12**: 197–202

Nakae D, Mizumoto Y, Kobayashi E, Noguchi O and Konishi Y (1995) Improved genomic/nuclear DNA extraction for 8-hydroxydeoxyguanosine analysis of small amounts of rat liver tissue. *Cancer Lett* **97**: 233–239

Nakagawa H, Kaneko S, Shima H, Inamori H, Fukuda H, Kominami R, Sugimura T and Nagao M (1997) Induction of minisatellite mutation in NIH3T3 cells by treatment with the tumor promoter okadaic acid. *Proc Natl Acad Sci USA* **94**: 10813–10816

Oberley TD and Oberley LW (1997) Antioxidant enzyme levels in cancer. *Histol Histopathol* **12**: 525–535

Okada F, Hosokawa M, Hasegawa J, Ishikawa M, Chiba I, Nakamura Y and Kobayashi H (1990) Regression mechanisms of mouse fibrosarcoma cells after in vitro exposure to quercetin: diminution of tumorigenicity with a corresponding decrease in the production of prostaglandin E2. *Cancer Immunol Immunother* **31**: 358–364

Okada F, Hosokawa M, Hamada J-I, Hasegawa J, Kato M, Mizutani M, Ren J, Takeichi N and Kobayashi H (1992) Malignant progression of a mouse fibrosarcoma by host cells reactive to a foreign body (gelatin sponge). *Br J Cancer* **66**: 635–639

Okada F, Hosokawa M, Hamada J-I, Hasegawa J, Mizutani M, Takeichi N and Kobayashi H (1993) Progression of a weakly tumorigenic mouse fibrosarcoma at the site of early phase of inflammation caused by plastic plates. *Jpn J Cancer Res* **84**: 1230–1236

Okada F, Hosokawa M, Hasegawa J, Kuramitsu Y, Nakai K, Yuan L, Lao H, Kobayashi H and Takeichi N (1994) Enhancement of in vitro prostaglandin E2 production by mouse fibrosarcoma cells after co-culture with various anti-tumour effector cells. *Br J Cancer* **70**: 233–238

Pitot HC (1986) The natural history of neoplastic development: progression. In *Fundamentals of Oncology*. Pitot HC (ed.), pp.163–200. Marcel Dekker: New York

Pitot HC (1989) Progression: the terminal stage in carcinogenesis. *Jpn J Cancer Res* **80**: 599–607

Rosin MP, Anwar WA and Ward AJ (1994) Inflammation, chromosomal instability, and cancer: the schistosomiasis model. *Cancer Res* **54**: 1929s–1933s

Schraufstatter I, Hyslop PA, Jackson JH and Cochrane CG (1988) Oxidant-induced DNA damage of targent cells. *J Clin Invest* **82**: 1040–1050

Schreck R and Baeuerle PA (1991) A role for oxygen radicals as second messengers. *Trends Cell Biol* **1**: 39–42

Shacter E, Beecham EJ, Covey JM, Kohn KW and Potter M (1988) Activated neutrophils induce prolonged DNA damage in neighboring cells. *Carcinogenesis* **9**: 2297–2304

Shibutani S, Takeshita M and Grollman AP (1991) Insertion of specific bases during DNA synthesis past the oxidation-damaged base 8-oxodG. *Nature* **349**: 431–434

Shimoda R, Nagashima M, Sakamoto M, Yamaguchi N, Hirohashim S, Yokota J and Kasai H (1994) Increased formation of oxidative DNA damage, 8-hydroxydeoxyguanosine, in human livers with chronic hepatitis. *Cancer Res* **54**: 5171–5172

*© Cancer Research Campaign 1999*

Southern EM (1975) Detection of specific sequences among DNA fragments separated by gel electrophoresis. *J Mol Biol* **98**: 503–515

Suemizu H, Yoshimura S, Takeichi N and Moriuchi T (1994) Decreased expression of liver glutathione peroxidase in Long–Evans Cinnamon mutant rats predisposed to hepatitis and hepatoma. *Hepatology* **19**: 694–700

Suzuki K, Nakata T, Seo HG, Miyazawa N, Sugiyama T and Taniguchi N (1991*a*) Differential expression of Mn- and Cu,Zn-superoxide dismutases in various tissues of LEC rats. In *The LEC Rat*. Mori M, Yoshida MC, Takeichi N and Taniguchi N (eds.), pp.142–148. Springer-Verlag: Tokyo

Suzuki S, Takada T, Sugawara Y, Muto T and Kominami R (1991*b*) Quercetin induces recombinational mutations in cultured cells as detected by DNA fingerprinting. *Jpn J Cancer Res* **82**: 1061–1064

Tabor E and Kobayashi K (1992) Hepatitis C virus, a causative infectious agent of non-A, non-B hepatitis: prevalence and structure-summary of a conference on hepatitis C virus as a cause of hepatocellular carcinoma. *J Natl Cancer Inst* **84**: 86–90

Takada T, Suzuki S, Sugawara Y, Kominami R, Arakawa M, Niwa O and Yokoro K (1992) Somatic mutation during metastasis of a mouse fibrosarcoma line detected by DNA fingerprint analysis. *Jpn J Cancer Res* **83**: 165–170

Taniguchi N (1992) Clinical significances of superoxide dismutases: changes in aging, diabetes, ischemia, and cancer. *Adv Clin Chem* **29**: 1–59

Thein SL, Jeffreys AJ, Gooi HC, Cotter F, Klint J, O'Cornor NTJ, Weatherall DJ and Wainscoat JS (1987) Detection of somatic changes in human cancer DNA by DNA fingerprint analysis. *Br J Cancer* **55**: 353–356

Weitzman SA and Gordon LI (1990) Inflammation and cancer: role of phagocyte-generated oxidants in carcinogenesis. *Blood* **76**: 655–663

Willard HF (1990) Centromeres of mammalian chromosomes. *Trends Genet* **6**: 410–416

Yamashina K, Miller BE and Heppner GH (1986) Macrophage-mediated induction of drug-resistant variants in a mouse mammary tumor cell line. *Cancer Res* **46**: 2396–2401

Yoshimura S, Komatsu N and Watanabe K (1980) Purification and immunohistochemical localization of rat liver glutathione peroxidase. *Biochim Biophys Acta* **621**: 130–137

Young MRI, Okada F, Tada M, Hosokawa M and Kobayashi H (1991) Association of increased tumor cell responsiveness to prostaglandin E2 with more aggressive tumor behavior. *Invasion Metastasis* **11**: 48–57

Yu MW, You SL, Chang AS, Lu SN, Liaw YF and Chen CJ (1991) Association between hepatitis C virus antibodies and hepatocellular carcinoma in Taiwan. *Cancer Res* **51**: 5621–5625

*© Cancer Research Campaign 1999*
